# Single Cell Droplet-Based Efficacy and Transcriptomic Analysis of a Novel Anti-KLRG1 Antibody for Elimination of Autoreactive T Cells

**DOI:** 10.21203/rs.3.rs-4745216/v1

**Published:** 2024-09-08

**Authors:** Tania Konry, Matthew Sulllivan, Aldo Rozzo, Antonio Ward, Patricia Rao, Dulce Soler-Ferran, Steven Greenberg

**Affiliations:** Northeastern University; Northeastern University; Abcuro, Inc.; Abcuro, Inc.; Abcuro, Inc.; Abcuro, Inc.; Abcuro, Inc.

**Keywords:** Immunotherapy, NK cell biology, Droplet sorting, Single-cell droplets, Immuno-oncology, Transcriptomic sequencing, Cancer cell biology

## Abstract

Progress in developing improvements in the treatment of autoimmune disease has been gradual, due to challenges presented by the nature of these conditions. Namely, the need to suppress a patient’s immune response while maintaining the essential activity of the immune system in controlling disease. Targeted treatments to eliminate the autoreactive immune cells driving disease symptoms present a promising new option for major improvements in treatment efficacy and side effect management. Monoclonal antibody therapies can be applied to target autoreactive immune cells if the cells possess unique surface marker expression patterns. Killer cell lectin like receptor G1 (KLRG1) expression on autoreactive T cells presents an optimal target for this type of cell depleting antibody therapy. In this study, we apply a variety of in vitro screening methods to determine the efficacy of a novel anti-KLRG1 antibody at mediating specific natural killer (NK) cell mediated antibody-dependent cellular cytotoxicity (ADCC). The methods include single-cell droplet microfluidic techniques, allowing timelapse imaging and sorting based on cellular interactions. Included in this study is the development of a novel method of sorting cells using a droplet-sorting platform and a fluorescent calcium dye to separate cells based on CD16 recognition of cell-bound antibody. We applied this novel sorting method to visualize transcriptomic variation between NK cells that are or are not activated by binding the anti-KLRG1 antibody using RNA sequencing. The data in this study reveals a reliable and target-specific cytotoxicity of the cell depleting anti-KLRG1 antibody, and supports our droplet-sorting calcium assay as a novel method of sorting cells based on receptor activation.

## INTRODUCTION

Autoimmune diseases encompass a wide range of conditions in which the immune system attacks normal, healthy tissues. This can arise from a variety of mechanisms in which self-antigens become recognized as foreign by immune cells. These diseases can be severely debilitating and typically are irreversible and can significantly decrease a patient’s quality of life. Untreated, many autoimmune diseases can be completely incapacitating, and in some cases fatal. Autoimmune disease may be difficult to treat due to the constant reinforcement of autoreactive immune cells recognizing healthy tissues as foreign, promoting the growth and expansion of these cells in a continuous feedback loop (1). Traditional treatment approaches focus on systemic suppression of inflammation to prevent autoreactivity. While effective at mitigating symptoms, this approach results in adverse symptoms, including increased susceptibility to infectious disease and certain cancers (2). Additionally, some treatment options can induce organ damage with long-term use, such as renal toxicitiy from nonsteroidal anti-inflammatories (NSAIDS) or neurotoxicity from biologics (2). These approaches are not disease modifying, and require lifelong treatment to manage symptoms. Specifically eliminating autoreactive immune cells might provide a highly effective treatment of autoimmune conditions that does not require the broad spectrum immune suppression of traditional treatments (3, 4).

To target treatment specifically to autoreactive immune cells, these cells must be identified based on unique features. KLRG1 has been shown to be expressed in particular on CD8^+^ cytotoxic T cells associated with autoimmune activity (5–7). Abcuro, Inc. has designed a cell depleting monoclonal antibody, specific for KLRG1 as a potential treatment strategy for certain autoimmune diseases in which KLRG1^+^ CD8^+^ cells have been identified as mediators of pathology. Depleting anti-KLRG1 antibodies bind to autoimmune cytotoxic T cells and target them for elimination by other immune cells and by complement, using mechanisms such as NK cell mediated antibody-dependent cellular cytotoxicity (ADCC) or macrophage mediated antibody-dependent cellular phagocytosis (ADCP), or complement dependent cytotoxicty (CDC). Autoreactive T cells are major drivers of autoimmune conditions, and targeted elimination of these cells can be enough to induce tolerance in the patient (8).

In this study, we assessed the in vitro activity of an anti-KRLG1-based immune cell depletion strategy in traditional bulk testing approaches and at the single cell level. We utilized a unique microfluidic droplet system to observe cytotoxicity and cellular interactions, with incorporation of a droplet sorting platform for additional downstream analysis. Cells expressing and lacking KLRG1 were combined with an anti-KLRG1 cell-depleting antibody and co-encapsulated with NK cells in our microfluidic cell droplet array device to observe interactions and killing through timelapse microscopy. The droplet sorting platform was then used to sort and isolate NK cells based on their ability to recognize and respond to anti-KLRG1 antibody binding, using a novel calcium-signalling based sorting approach. Sorted NK cells were analyzed with transcriptomic sequencing to observe what cellular characteristics may influence the efficacy of this treatment approach. Our results display promising efficacy of this anti-KLRG1 antibody through unique metrics, and describe a novel approach that can be utilized to further understand and enhance the cellular response to immunotherapies such as this.

## METHODS

### Device Fabrication and Use

Devices were fabricated in-house using previously described methods.(9) Briefly, devices were made from PDMS using photocrosslinked silicon wafers as a mold, and bonded to glass slides. Droplets are generated on-chip from a cell suspension in media and FC-40 oil (3M, Maplewood, Minnesota), creating aqueous-in-oil emulsions. Cell suspensions and oil are delivered to the device using syringe pumps (Harvard Apparatus, Holliston, MA). Droplets are loaded with approximately 1–3 of each cell type on encapsulation and held in a 4000 docking site array for imaging.

### Cell Culture

Parental and KLRG1 transfected CHO-K1 cell lines were provided by Abcuro, Inc. Briefly, one stable cell line using CHO-K1 as the host was established to overexpress human KLRG1. The full length cDNA of human KLRG1 was cloned into pLVX plasmid. Then lentivirus production was performed by co-transfecting the pLVX and helper plasmids using commercial packaging kit (Takara, Cat#631275) in 293T cells. Virus supernatant was collected at both 48 h and 72 h after transfection. Then CHO-K1 cells (ATCC) were used for lentivirus infection with polybrene (Sigma, Cat# H9268). After 72 h infection, cells were selected using puromycin(Gibco, Cat#A11138–02) at 8 μg/ml and then FACS sorting were used for single clone selection with high KLRG1 expression level. CHO cells were grown in RPMI 1640 (ATCC) with 10% ultra-low IgG FBS and 1% antibiotic mix (Gibco, Waltham, MA). Peripheral blood NK cells (≥85% CD56 positive) were purchased frozen from Stemcell Technologies and thawed in the same media used for CHO cells, with the addition of 15 ng/mL IL2 (Peprotech). NK cells were rested overnight after thawing prior to running experiments. Human CD57^+^CD8^+^ T cells were isolated from isolated human CD8^+^ T-cells which were in turn isolated from 125 mL leukopaks from healthy human donors utilizing EasySep human CD8^+^ T cell isolation kit from StemCell. CD57^+^ CD8^+^ T cells were further isolated from total isolated CD8^+^ T cells with CD57 Microbeads, Human from Miltenyi Biotec. Manufacturer’s instructions were followed for the cell isolations. Purities greater than 85% for the three isolated populations were confirmed by flow cytometry. The vast majority of CD57^+^ CD8^+^ T cells expressed KLRG1 and hence, they were used as KLRG1^+^ CD8^+^ T cells. KLRG1^−^CD8^+^ T cells were used also as negative controls. These cells were prepared from total CD8^+^ T cells stained with anti-KLRG1-PE antibody (BioLegend; ~1 μl/million cells) followed by isolation with Anti-PE MicroBeads UltraPure (Miltenyi Biotec) per manufacturer recommendations, yielding two populations: KLRG1^+^ CD8^+^ T cells and KLRG1^−^ CD8^+^ T cells.

Antibody treatments were added to cell suspensions at 10 μg/mL immediately before droplet generation or at time 0 in plate assays. Anti-KLRG1 antibody Ulviprubart (ABC008) is an afucosylated humanized human IgG1 antibody in clinical trials (10–12) and it was provided by Abcuro, Inc. Afucosylated human IgG1 isotype control, Anti-b-Gal-hIgG1fut from InvivoGen was used for the negative control.

### Fluorescent Microscopy

Devices were imaged on an Axio Observer microscope equipped with an automated stage and incubation chamber for maintaining cells at 37°C and 5% CO_2_. Target cells (CD8^+^ T cells and CHO-K1) were labeled with calcein AM (Invitrogen, Waltham, MA) as a live stain, and ethidium homodimer III (Biotium, Freemont, CA) was added to droplets to image dead cells. Separate microfluidic devices were loaded for each condition, and imaged at 20X magnification every 15 minutes for 8 + hours. Cell death was determined by a combination of morphology and viability dyes.

### Plate Cytotoxicity Assay

NK cells and CHO-K1 cells were prepared as done in the droplet co-encapsulation experiments, described above. CHO cells were labeled with calcein green AM for 30 minutes and washed prior to plate loading. 30,000 target CHO cells were loaded per well, with NK cells loaded at 0:1, 1:1, 2:1 and 5:1 effector to target ratios. Plates were incubated at 37° C and 5% CO_2_ for 5 hours, then were spun down and supernatant removed to remove calcein released from dead cells. Fluorescence was then measured with a ThermoFisher VarioSkan LUX plate reader using 490 excitation / 520 emission.

### Droplet Sorting and Collection

For the droplet sorting experiments, we used our own sorting platform to recognize and sort fluorescent events. This platform utilizes a microfluidic device to generate and sort droplets via diaelectric field. Fluorescence is detected by PMTs (Hamamatsu Photonics, Hamamatsu City, Japan) when droplets reach the sorting junction, which feeds back to a control unit (National Instruments, Austin, TX). Using LabVIEW software (National Instruments), fluorescent signal is continuously monitored, and a threshold is set to determine fluorescent peaks. Peaks over threshold automatically trigger an electric impulse sent to the device, amplified via a high-voltage amplifier (Advanced Energy, Denver, CO), which generates the droplet-sorting field.

For this experiment, calcium signaling was used to sort NK cells activated through fragment crystallizable gamma receptor IIIa (FcgRIIIa) binding. Prior to the experiment, NK cells were incubated for 1 hr with 5 μM Fura-10 AM ratiometric calcium dye (AAT Bioquest, Sunnyvale, CA). KLRG1^−^ CHO-K1 cells were incubated with a-KLRG1 Ab for 30 minutes, and washed twice immediately prior to droplet generation. Droplets were generated on-chip, encapsulating NK and CHO cells at 1:1 and mixing for 1 minute prior to reaching the sorting array. Calcium mobilization occurs upon FcR binding and NK cells with elevated calcium levels produce a fluorescent signal spike, and are automatically sorted based on threshold values set at the start of the experiment. Positively selected and unselected droplets are collected separately, the emulsion is disrupted through gentle pipetting and centrifugation. Cells are then processed with the RNeasy mini kit (QIAGEN, Venlo, Netherlands), and collected RNA is stored at −80°C. RNA is sent to Novogene (Beijing, China) for transcriptomic sequencing and bioinformatic analysis. NK and CHO co-encapsulations were performed in duplicate, and a CHO-alone control sample was additionally sequenced for identification of hamster sequences mapping to human genes.

## RESULTS

### Bulk and Single-Cell Visualization of Anti-KLRG1 Antibody ADCC Activity

To ensure that the anti-KLRG1 antibody mediated immune cell killing exclusively through KLRG1 binding of the target cell, we utilized CHO-K1 cells transfected to express KLRG1. Expression of KLRG1 was confirmed through flow cytometry, which revealed high levels of consistent expression in transfected CHO cells (Supplemental Fig. 1). We explored 4 conditions in these studies; KLRG1^−^ parental CHO cells and KLRG1^+^ transfected CHO cells with either the anti-KLRG1 antibody (a-KLRG1 Ab) or an afucosylated isotype-control antibody (Control Ab). NK cells were utilized as the effector cells for all experiments. Antibody concentration ranges to use were determined using titration in bulk culture and measured with flow cytometry (Supplemental Fig. 2). We began with a plate-based cytotoxicity assay to assess the ability of a-KLRG1 Ab to induce ADCC mediated killing at multiple effector-to-target ratios (Fig. 2). We found that the presence of a-KLRG1 Ab increased killing in KLRG1^+^ CHO at all E:T ratios, but only at the 5:1 ratio was this increase statistically significant. The anti-KLRG1 antibody did not seem to affect the viability of Parental CHO-K1 cells, nor did it have any notable direct effects on KLRG1^+^ CHO cells in the absence of effector cells (Fig. 2D).

To measure the activity of the a-KLRG1 Ab with higher precision, the cytotoxicity assay was evaluated in the single-cell droplet platform. Fluorescent microscopy, allows the generation of timelapse images to record cytotoxicity and cell-cell contact (Supplemental Movie 1). A minor increase in cytotoxicity observed using KLRG1^+^ CHO target cells at an E:T of 1:1. (Supplemental Fig. 3A). This NK cytotoxicity was notably higher in the a-KLRG1 Ab treated KLRG1 + CHO cells at a 2:1 E:T ratio (Fig. 3B,D). The anti-KLRG1 antibody again did not appear to impact killing of Parental CHO cells. The 5:1 condition was not repeated in droplets due to volume and encapsulation rate limitations.

We also observed differences in the interaction kinetics between NK and CHO cells across the different conditions. The presence of the a-KLRG1 Ab resulted in a significant increase in contact of NK and high-KLRG1 CHO cells. NK from different donors were used for the KLRG1^+^ CHO experiments (Supplemental Fig. 3B), which may contribute to differences seen between the different CHO cell populations.

### Single-Cell Visualization of Anti-KLRG1 Antibody Activity Towards Human CD8^+^ T Cells

Next, we studied the ability of the anti-KLRG1 antibody to induce ADCC mediated killing of KLRG1-expressing CD8 + T cells, the target cell of interest for this therapy. CD8^+^ T cells were sorted based on CD57^+^ expression, a surface marker strongly co-expressed on a subset of KRLG1^+^CD8^+^ T cells. We utilized the single-cell droplet platform for these experiments. Viability of T cells was observed over 2 hours, as these cells were prone to spontaneous death in droplets after this time window. We studied NK cell interactions with CD57^+^ and KLRG1^−^ CD8^+^T cells with either the a-KLRG1 Ab or isotype control. We found that the a-KLRG1 Ab elicited a similar response as seen with the CHO cells (Fig. 4), however the best killing was observed at 1:1 E:T ratios. At the 2:1 ratio, killing of CD8^+^CD57^+^ T cells increased in both treated and control conditions (Supplemental Fig. 4). Additionally, no differences in contact affinity of NK cells and T cells were observed between control conditions, however a slight increase in contact time was observed in CD8^+^CD57^+^ T Cells with a-KLRG1 Ab (Supplemental Fig. 4F).

Based on these data, it appears a single NK cell is sufficient to kill a KRLG1^+^CD8^+^ T cell treated with a-KLRG1 Ab. Increased E:T ratios do not improve ADCC mediated cytotoxicity in the droplets, however it does seem to increase spontaneous killing of KLRG1 + T cells, as observed in the isotype control condition (Supplemental Fig. 4). NK cell viability was consistent across all treatment conditions, indicating anti-KLRG1 antibody produces no cytotoxicity towards NK cells themselves (Supplemental Fig. 4E).

### Droplet Sorting for Evaluation of Transcriptomic Signatures Influencing NK Cell Activation By Anti-KLRG1 Antibody

**Figure 1:**
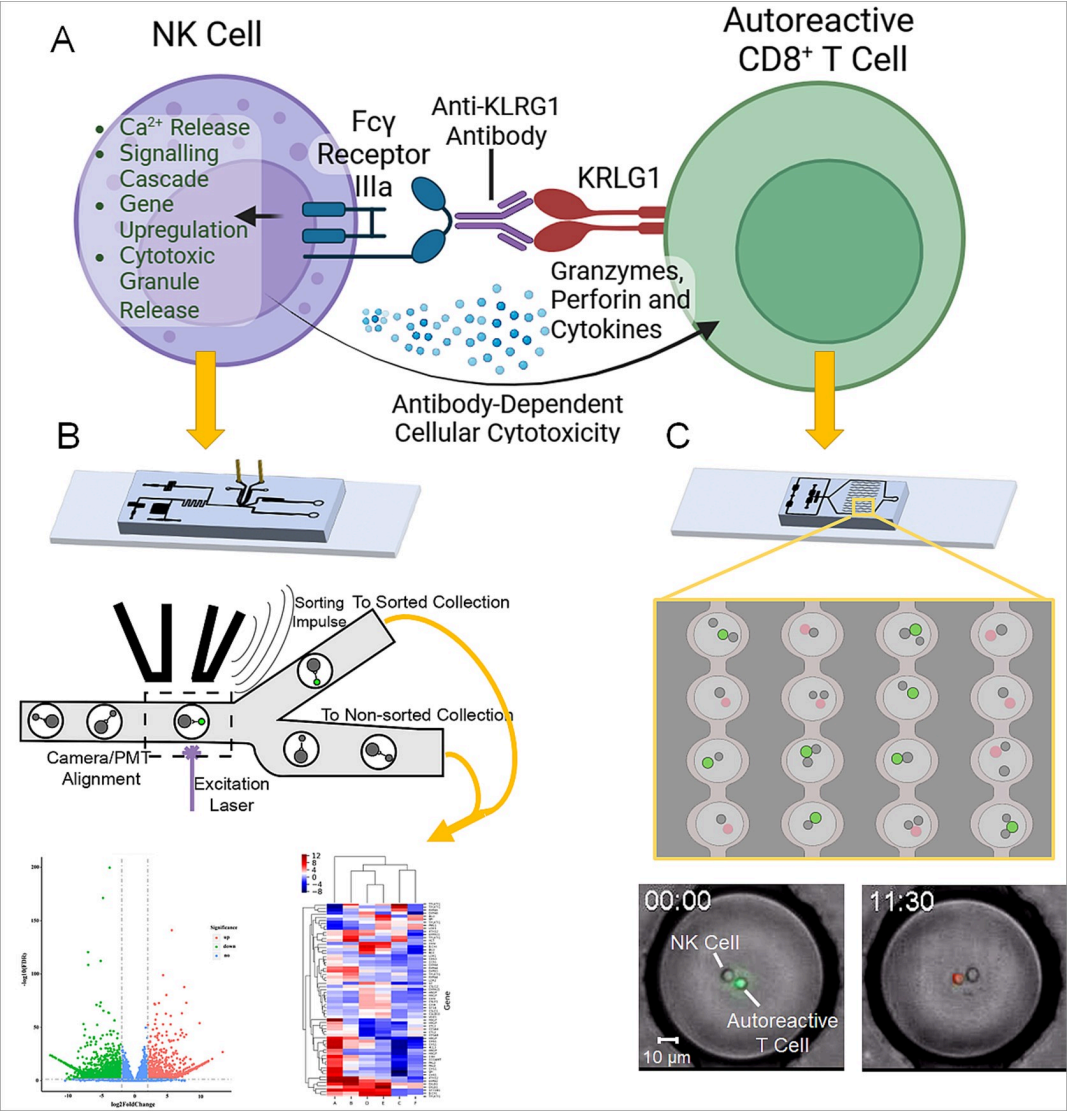
Graphical abstract of presented research. **(A)** Drawing depicting a simplified representation of the ADCC response mediated by NK cells and the anti-KLRG1 antibody. **(B)** Overview of droplet sorting process, using the calcium release in response to CD16 binding to sort based on antibody binding. RNA is extracted from sorted cells for transcriptomic analysis. **(C)** Overview of timelapse imaging in microfluidic droplets to observe the activity of a-KLRG1 Ab at the single-cell level.

To develop an understanding of the cellular factors involved in NK cell response, or lack of response to the a-KLRG1 Ab, we next sought to sort out NK cells based on their FcgRIII binding and response to the a-KLRG1 Ab also bound to KLRG1- expressing cells. To effectively sort NK cells based on recognition of an antibody-labelled cell, they must be combined in droplets and quickly sorted. Our platform has been demonstrated to reliably sort droplets based on fluorescent signal (13). For this study, combined NK cells treated with a ratiometric calcium dye and KLRG1^+^ CHO cells pre-treated with the a-KLRG1 Ab in droplets. While Ca^2+^ transport kinetics can be variable, the need for Ca^2+^ release as part of signaling cascades is highly conserved for receptor function, including FcgRs and several other immune receptors (14). In our Fluorescence-Assisted Droplet Sorting (FADS) protocol, cellular Ca^2+^ levels create a corresponding change in fluorescent intensity. Basal Ca^2+^ level differences between cell populations will be observable by the measured fluorescent intensity, and we adjust our sorting thresholds accordingly to remain above cell baseline levels (Supplemental Movie 2). Release of Ca^2+^ via a cell signaling event produces a dramatic increase in fluorescent intensity, allowing us to visually verify that sorting is based on cell stimulation, presumably through receptor binding. This enables us to reliably sort cells based on the release of Ca^2+^, despite the mentioned variation across cells and cell types. To apply Ca^2+^ -based sorting to this, we used NK cells combined with anti-KRLG1 antibody treated KRLG1^+^ CHO cells in hopes of isolating cells based on Fc receptor binding. The transfected CHO cell was chosen as a target due to its consistent and high expression of KLRG1, increasing likelihood of NK cell interaction. Since this antibody activates ADCC mediated cytoxicity, the first step should be binding of the CD16 Fc gRIIIa receptor to anti-KLRG1 Ab. Successful receptor binding would produce an intracellular calcium release as part of the signaling cascade (14, 15). To ensure calcium peaks are not missed, NK and target cells are combined directly on chip, and given approximately 30 seconds to interact prior to reaching the device’s sorting junction. At this junction, cells expressing increased calcium levels were automatically sorted to one outlet, while all other droplets flowed to a separate outlet, as displayed in Fig. 1B. From the calcium signal positive and negative cells, we isolated mRNA for transcriptomic analysis. CHO cells were also directly submitted for sequencing to screen for any hamster sequences erroneously attributed to human gene hits that may confound our transcriptomic analysis.

Our transcriptomic sequencing found subtle transcriptomic differences between the Calcium-positive (sorted) and negative (unsorted) populations. Overall, 2204 genes were upregulated and 2085 genes were downregulated in the population displaying calcium release (Fig. 5A). When considering the most differentially expressed genes between both populations, many of these genes are involved in mediating transcription and translation (Fig. 5B) (16–19). Of particular interest, *CD69* and *CD244*, which both serve as markers of NK cells activation and can induce cytotoxicity towards target cells, are significantly higher in the Calcium Positive population (16–21). The GO analysis of the most significantly upregulated terms in calcium positive cells included several terms associated with effector cell activity (Fig. 5C). These terms included adaptive immune response, antigen binding and cytokine activity. These terms infer a high level of cytotoxic activity in the calcium positive NK cells. In the GO analysis of genetic factors more highly expressed in the calcium negative population, terms related to metabolic activity, especially oxidative phosphorylation, associated with resting NK cells, were most abundant. The highest expressing genes in the calcium-positive NK cells are listed in Supplemental Table 1.

To further characterize the differences between cells, we looked at differences in individual expression of genes between the two populations, highlighting genes related either to the NK cell calcium signaling pathway, or to NK cell cytotoxic activity. (Fig. 6). All genes presented were confirmed to be negative in the CHO control sample. Due to limited number of replicates, no expression differences were statistically significant between groups, however interested trends in expression levels were observed. Amongst the genes involved in calcium signaling, *ZAP70* and *SYK* were expressed notably higher in the calcium-positive NK cells; these two kinases are known to be associated with signaling through CD16 (FcgRIIIa) in NK cells (22, 23). *LCK* and *FCERG1* expression were also slightly higher in the calcium positive NK cells. *S100A4, ADAM17* and *CD247* were all more highly expressed in the calcium negative population. CALM1 was also approximately twofold higher in the calcium negative cells.

Of the genes observed involved in NK cell maturity and cytotoxic activity, *FCGR3A* (CD16) and *HAVCR2* were expressed almost 2 fold higher in calcium positive cells, while *NCAM1* (CD56) was higher in the calcium negative cells. In addition to the direct effect of CD16 expression on antibody binding, these expression levels indicate a more mature and active phenotype in the calcium positive cells. Interestingly, *GZMA* and *GZMB* (granzymes A and B), *PRF1* (perforin), *FASLG* (Fas ligand) and *TNFSF10* (TRAIL) were also all more highly expressed in the calcium positive cells. Granzymes and perforin directly are responsible for the target cell killing following CD16 activation, indicating the NK cells producing a calcium response to Ulviprubart would also be more able to kill their target cells (24, 25). *KLRC1* (NKG2A) and *KLRD1* (CD94) were also both higher in calcium positive cells. Overall, the expression patterns of calcium positive and negative cells coincide with the expectations of NK cells that would or would not activate an ADCC response, supporting the accuracy of the droplet sorting platform.

## DISCUSSION

**Figure 2:**
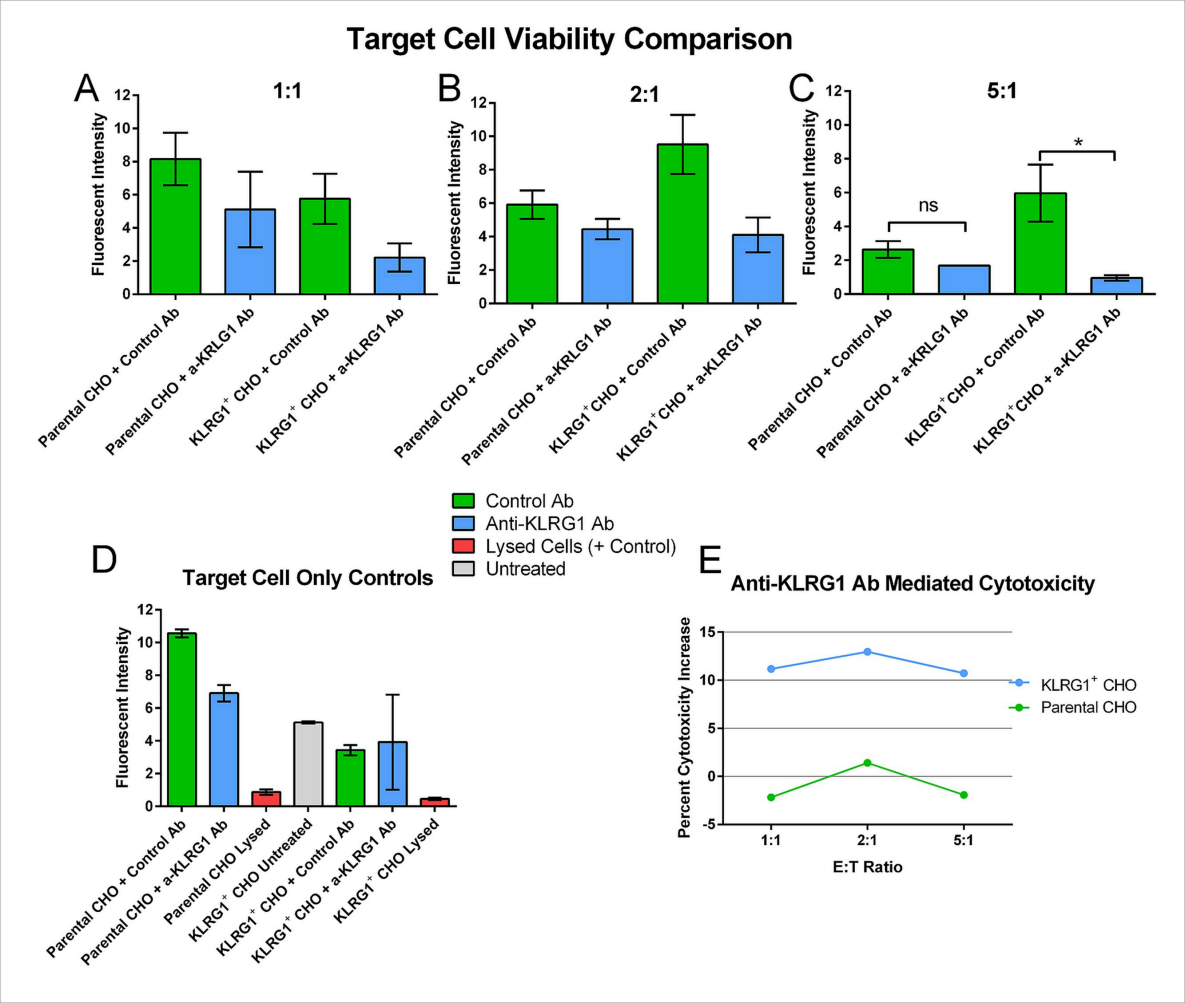
Specificity of the anti-KRLGI antibody (a-KLRG1 Ab) toxicity towards KLRG1^+^ CHO cells via plate assay compared to isotype control antibody (Control Ab). A-C) Viability of CHO cells as measured by calcein fluorescence in various treatment conditions with **(A)** 1:1, **(B)** 2:1 and **(C)** 5:1 Effector to Target ratios. **(D)** Viability of CHO cells in the absence of NK cells to observe direct effects of anti-KLRG1 or isotype control antibodies. **(E)** Percent increase in cytotoxicity of each condition, normalized to NK-free control for each treatment. Error bars represent Standard Error. * represents *p* < 0.05.

**Figure 3:**
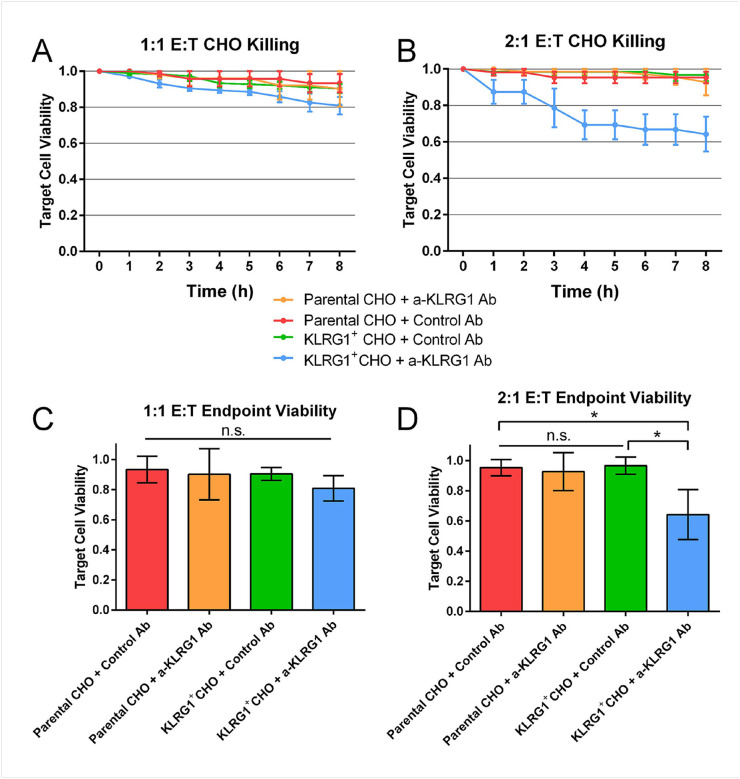
Single-cell droplet cytotoxicity data of CHO cells co-encapsulated with NK. Droplets were imaged every 15 minutes for 8 hours. **(A)** Cytoxicity data of 1:1 effector:target co-encapsulations. Parental = 74, KLRG1= 116 cells. **(B)** Cytoxicity data of 2:1 co-encapsulations. Parental = 62, KLRG1 = 128 cells. **(C)** Bar graph depicting CHO cell viability at 1:1 co-encapsulation at the end of 8 hours. **(D)** Bar graph depicting CHO cell viability at 2:1 co-encapsulation at the end of 8 hours. * = p value < 0.05.

In this study, we have utilized both traditional and novel in vitro methodologies to assess the activity of a cell depleting anti-KRLG1 antibody on NK cell mediated ADCC, as a method to eliminate autoreactive CD8^+^ T cells. Utilizing a transfected KLRG1^+^ CHO cell, we first observed the specificity of this antibody for mediating ADCC exclusively through KLRG1 recognition. Our plate assay confirmed cytotoxicity increases were only observed in the KLRG1^+^ CHO with the anti-KLRG1 Ab (Fig. 2). Additionally, no significant increase in target cell killing by the anti-KLRRG1 Ab was observed in the absence of NK cells, indicating no direct toxicity by the antibody binding (Fig. 2D). These results were also confirmed by a flow cytometry ADCC assay (Supplemental Fig. 2) as well as via our single-cell droplet platform (Fig. 3). Both plate and single-cell assays displayed a significant increase in cytoxicity in higher E:T ratios. Additionally, we observed a significant increase in effector-target contact in the KLRG1^+^ CHO with the anti-KLRG1 antibody, but not in the KLRG1^−^ CHO (Supplemental Fig. 1). This is presumably due to the high-affinity binding of CD16 to a-KLRG1 Ab.

**Figure 4:**
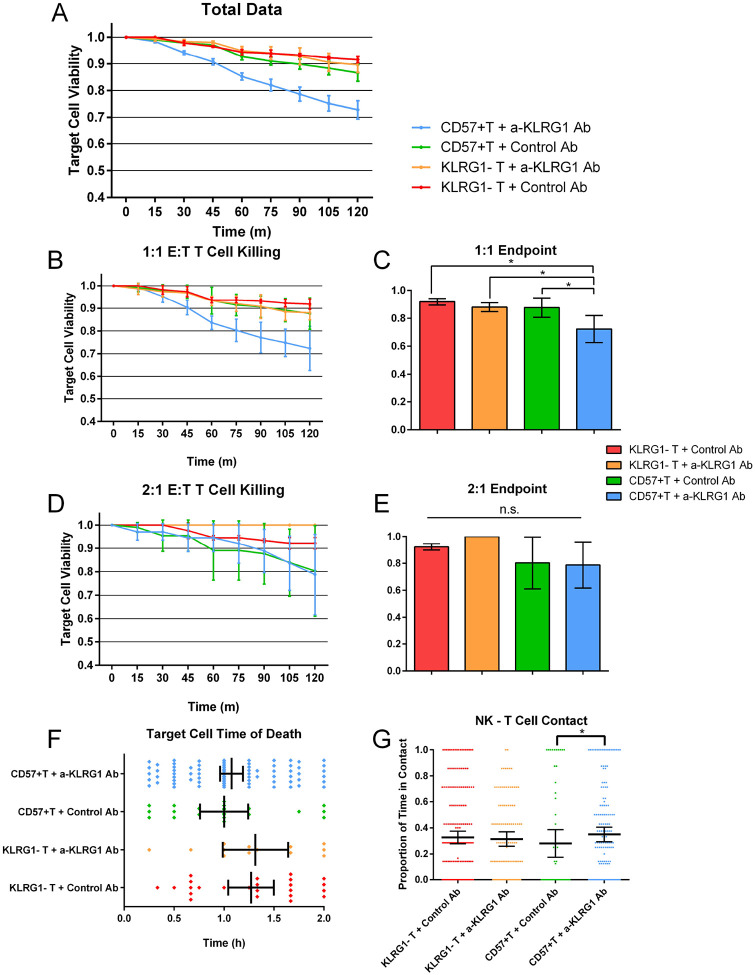
Single-cell droplet observations of NK cell cytotoxicity towards CD8^+^ T cells over 2 hours. **(A)** CD8^+^ T cell viability over time. N: CD57^+^ T + Treatment = 361, CD57^+^ T + Isotype Control = 213, KRLG1^−^ T + Treatment = 244, KRLG1^−^ T + Isotype Control = 361 target cells. **(B)** Bar graph representation of T cell viability at 2-hour endpoint. * = *p* < 0.05.

We next tested this antibody on KLRG1^+^ versus KLRG1^−^ CD8^+^ T cells using our single-cell droplet microfluidic array. We observed specific killing only towards the KLRG1 expressing T cells (Fig. 4), as well as an increase in effector-target contact (Supplemental Fig. 4F). We did not observe and increase in killing with increased E:T ratios, which indicates the 1:1 NK to T cell ratio as the most efficient. This result is promising for clinical application, as ratios of NK cells to KLRG1^+^ CD8^+^ T cells in circulation are roughly 1:1 or lower in adults (26–28). As with the CHO cells, the KLRG1^−^ T cells were unaffected by the a-KLRG1 Ab, suggesting no spontaneous toxicity. Additionally, the NK cell viability was also unaffected, indicating the antibody does not induce significant levels of fratricide killing between NK cells (Supplemental Fig. 4E).

**Figure 5:**
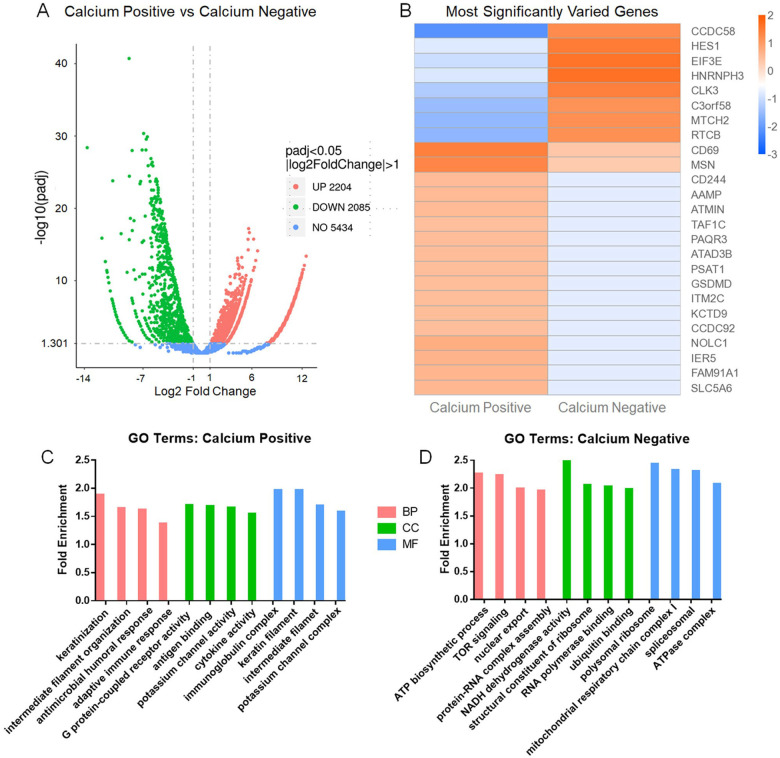
Transcriptomic data of NK and KLRG1 expressing CHO cells sorted based on intracellular calcium release. Sorted NK cells possessed elevated calcium levels, while unsorted showed no change from baseline. **(A)** Volcano plot of gene expression differences between calcium positive and negative cells. **(B)** Heatmap representing the 25 genes most significantly differing between Calcium Positive and Calcium Negative populations. **(C)** Gene Ontology (GO) Analysis of terms related to upregulated genes in the sorted cells. **(D)** GO Analysis of terms related to upregulated genes in the Calcium Negative, unsorted cells. BP = Biological Processes, CC = Cellular Components and MF = Molecular Factors.

After establishing the ability of the a-KLRG1 Ab to elicit ADCC towards KLRG1^+^ cells, we next developed a protocol to sort NK cells based on response to the a-KLRG1 Ab using our droplet sorting platform. NK cells and a-KLRG1 Ab-bound CHO cells were paired on-chip, and a ratiometric calcium dye was utilized to identify cells with increased intracellular calcium. These cells presumably recognized the anti-KLRG1 Ab through CD16 receptor binding, and would be undergoing the first stages of the ADCC response when sorted, although the short time elapsed between receptor activation and collection of RNA probably permitted identification of only transcripts already present in the responding cells or those most rapidly induced after receptor triggering. We collected the sorted cells and used transcriptomic sequencing to validate this method, and potentially uncover genetic factors influencing anti-KLRG1 Ab recognition. Despite the short time lapse between receptor binding/triggering and sample collection we did observe variation between the calcium positive and calcium negative NK cell populations for expression of genes relevant to NK function (Fig. 5). Many of the most significantly different genes between the two populations tend to have inherently high expression levels in mammalian cells and may fluctuate greatly based on cell cycle (Fig. 5B) (16–19). They included various proteins involved in translation and transcription and may have represented the initial steps in transcription/translation associated with FcgR signaling.

**Figure 6:**
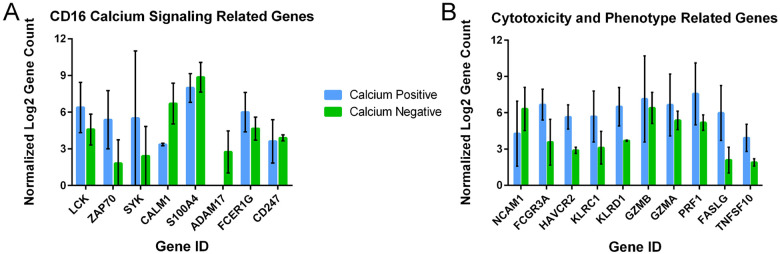
Bar graphs representing the log2 expression of normalized gene counts in Calcium Positive (blue) versus Calcium Negative (green) sorted cells. **(A)** Genes related to the CD16 signaling cascade and calcium release. **(B)** Genes related to NK cell cytotoxic activity and maturity/phenotype. Error bars represent SEM.

To further elucidate the differences between populations, we looked at several genes related to NK cell functionality and calcium signaling (Fig. 6).For the genes related to NK cell functionality, our findings supported droplet sorter’s capability to isolate NK cells based on CD16 activity (Fig. 6A). As expected, the calcium-positive NK cells had higher levels of CD16 (*FCGR3A*). Lower expression of *FCGR3A* (CD16) and higher expression of *NCAM1* (CD56) in the calcium negative population also suggest that the NK cells that did not recognize anti-KRLG1 antibody seem to possess an immature phenotype (Fig. 6B). Lower expression of *HAVCR2* (TIM3) further supports this observation, suggesting either an immature or downregulated phenotype (29, 30). Higher expression of CD244 and CD69 (Fig. 5B) also support a more active phenotype in the calcium positive NK group (20, 21). Of the genes associated with the calcium cascade (Fig. 6A), *ZAP70* and *SYK* had the most significant increase in expression in the calcium positive NK. Zap70 and Syk levels have been found to be highly correlated to NK cell activity and are triggered after CD16 activation (22, 23, 31, 32). Coinciding with this, Lck, which also had higher expression in the calcium positive cells, promotes Zap70 signaling, further reinforcing the importance of this pathway in the response of these NK cells to a-KLRG1 Ab binding (33). The integral membrane proteins FcεR1γ and CD247 are also important for NK cell calcium signaling, as they bind to CD16 and stabilize its activation (34). Higher expression of *FCER1G* in the calcium positive cells and minimal difference in *CD247* expression indicate FcεR1γ, associated with NK cells with strong cytotoxic effector function is also a key factor in the response to anti-KLRG1 antibody (35). ADAM17, S100A4 and Calmodulin are more highly expressed in the calcium negative NK population Calmodulin acts downstream of calcium signaling, responding to calcium concentrations to mediate further downstream signaling (36). S100A4 has been shown to attenuate signaling by binding CD16, and ADAM17 induces shedding of the CD16 receptor from the plasma membrane (24, 32). Therefore, the increased expression of these factors provides logical mechanisms reducing the NK cell response to the a-KLRG1 Ab in this population. This finding provides three potential characteristics of NK cells that will not respond to monoclonal antibody treatments through ADCC. In addition to observing genes that may influence the ability of an NK cell to respond to the a-KLRG1 Ab, we also observed variation in the expression of several genes related to cytotoxicity (Fig. 6B). Higher expression of *GZMA, GZMB* and *PRF1* suggest the calcium positive NK cells will be highly cytotoxic after CD16 activation (24, 37). Additionally, increased expression of the CD16-independent apoptosis-inducing ligands *FASLG* (Fas ligand) and *TNFSF10* (TRAIL) in the calcium positive NK cell population suggest they may have additional mechanisms available to kill target cells, promoted by the increased contact duration observed with anti-KLRG1 Ab treatment (Supplemental Figs. 2, 4).

To summarize, we observed consistent specificity of the anti-KLRG1 Ab for mediating elimination of target cells based on KLRG1 expression, with no direct toxicity in the absence of NK cells. We noted more significant effects in our single-cell platform than in a traditional plate assay, which correlates more accurately to the clinical expectations of Ulviprubart (10–12). These findings support the use of our single-cell droplet observation platform for sensitive and accurate analysis of treatment efficacy. We additionally developed a method for screening NK cells based on their ability to respond to antibody therapies by implementing our fluorescence-assisted droplet sorting platform with a fluorescent calcium assay, to sort NK cells based on activation of the CD16 receptor. Traditional cell-sorting approaches, such as flow cytometry-based sorting, are unable to sort based cells based on their functional interactions on another cell type. Droplets provide an optimal alternative due to their ability to encapsulate two cell types, and sort them based on fluorescent signal of either cell. Additionally, calcium release rapidly peaks and dissipates in a cell, requiring a platform that can both combine and sort to isolate cells based on calcium signaling (14, 38). This same methodology could be used to screen cells based on activation of other immune receptors, such as T cell receptors and chimeric antigen receptors (39, 40). Utilizing transcriptomic sequencing, the results reinforced the accuracy of this novel calcium-based droplet sorting assay by displaying functionally relevant phenotypic differences between sorted NK cell populations. We also presented several transcriptomic variations between the two populations suggesting potential genes influencing the CD16 response to this antibody. This study supports the efficacy of an anti-KLRG1 antibody for suppression of autoreactive CD8^+^ T cells with unique observations through our combined platform and presents a methodology for detailed in vitro screening of antibody treatments and other immunotherapies.

## Data Availability

Sequencing data is available in the National Insistute of Healths Genome Omnibus (GEO) unders accession number GSE267341.
